# Yoga Nidra: A Promising Complementary Therapy for Enhancing Cancer Care

**DOI:** 10.7759/cureus.70536

**Published:** 2024-09-30

**Authors:** Selvaraj Giridharan

**Affiliations:** 1 Oncology, Tawam Hospital, Al Ain, ARE

**Keywords:** cancer, qol, sleep, stress, yoga nidra

## Abstract

Cancer patients encounter not only the physical manifestations of their disease but also a range of psychological challenges, including anxiety, depression, and severe sleep disturbances. Research indicates that approximately 50% of cancer patients experience clinically significant levels of distress, which can adversely affect treatment outcomes and diminish their overall quality of life. Moreover, the side effects of treatments such as chemotherapy and radiotherapy frequently exacerbate mental health difficulties, creating an urgent need for effective alternative supportive interventions to ameliorate these symptoms. Yoga Nidra, or yogic sleep, may be particularly efficacious for patients with cancer undergoing intensive treatments, such as chemotherapy or radiotherapy, where physical limitations or psychological stress are more pronounced. As the medical community increasingly recognizes the value of holistic approaches to cancer care, there is a growing interest in integrating complementary therapies that address both the mental and physical aspects of health. One such therapy is Yoga Nidra, a guided meditation practice that has garnered attention for its potential to alleviate psychological distress and improve sleep quality in cancer patients. Given its non-invasive, cost-effective, and highly accessible nature, Yoga Nidra presents a promising approach for enhancing the overall well-being of individuals undergoing cancer treatment. This editorial examines the role of Yoga Nidra in cancer care, highlighting its distinct benefits and emerging evidence supporting its use as a complementary therapy.

## Editorial

What is Yoga Nidra?

Yoga Nidra, or yogic sleep, is a structured practice designed to achieve complete physical, mental, and emotional relaxation. The term "Yoga Nidra" originates from two Sanskrit words: "yoga”, which signifies union or focused awareness, and "nidra”, which refers to sleep. Although practitioners may appear to be asleep during Yoga Nidra, their consciousness operates at a deeper level of awareness, a state often described as "psychic sleep" or profound relaxation coupled with inner awareness [1]. This practice is particularly beneficial for cancer patients, as it directly addresses their psychological needs, including the management of anxiety, depression, and emotional fatigue, which are commonly associated with cancer and its treatment [2].

Yoga Nidra provides a unique method of engaging with subconscious and unconscious minds, promoting a sense of mental clarity and emotional relief. Furthermore, it is a deeply relaxing practice that combines guided meditation, body awareness, breathing techniques, and visualization. Unlike traditional yoga, which often requires physical postures, Yoga Nidra is performed while lying down in Shavasana (corpse pose), rendering it accessible to individuals experiencing fatigue, pain, or other physical limitations common in cancer patients.

Introduced to the public in the 1960s by Swami Satyananda Saraswati, Yoga Nidra facilitates spontaneous interaction with the subconscious and unconscious dimensions of the mind in the transitional state between sleep and wakefulness [3]. Yoga Nidra achieves this profound relaxation by directing attention inward and away from external stimuli, thereby promoting a unique state of conscious rest and self-exploration [4]. Research has investigated the potential benefits of Yoga Nidra in addressing neurological, neurocognitive, and psychological conditions as well as its broader effects on the human body. Evidence has demonstrated its efficacy in alleviating pain, improving cardiovascular health, enhancing immune function, and mitigating stress [5]. These advantages render Yoga Nidra suitable for a diverse range of individuals, from novices to experienced practitioners [6].

Benefits of Yoga Nidra for cancer patients

Yoga Nidra, a guided meditation practiced in Shavasana, is more appropriate for cancer patients than traditional Yoga because of its accessibility to individuals experiencing pain, fatigue, limited mobility, or treatment complications. The physical requirements of traditional Yoga may pose risks for patients with cancer, particularly those with compromised immune systems, bone fragility, or neuropathy. Yoga Nidra's emphasis on deep relaxation and stress reduction addresses the psychological issues prevalent among cancer patients such as anxiety, depression, and emotional fatigue. Its techniques, including guided imagery, breath awareness, and rotation of consciousness, aim to alleviate the nervous system and promote mental and emotional equilibrium, thus benefiting individuals with insomnia or sleep disturbances.

Yoga Nidra's minimal cost and flexibility make it an appealing complementary therapy, as it can be practiced in domestic or healthcare settings. Traditional yoga classes may incur expenses and may not be readily adaptable to home practice, especially for those with limited mobility. Yoga Nidra's structured yet customisable format allows sessions to range from 15-20 to minutes to one hour, contingent upon the patient's comfort and energy levels. Its guided nature facilitates the use of audio recordings, enabling patients to practice independently without requiring a live instructor, in contrast to traditional Yoga forms that require in-person guidance for safety and correct posture.

The evidence supporting Yoga Nidra in cancer care

Three randomized studies evaluated the efficacy of Yoga Nidra in patients with cancer and elucidated its potential benefits for sleep quality, stress reduction, and psychological well-being. Anand et al. examined the impact of Yoga Nidra on sleep quality in patients with cancer [7]. This study was conducted in two phases: an initial survey of 25 participants that assessed sleep quality using the Pittsburgh Sleep Quality Index (PSQI) and an intervention phase involving 19 participants with poor sleep quality. Participants underwent Yoga Nidra sessions, and their sleep quality was reassessed using a pre-test and post-test design. The results demonstrated a significant improvement in sleep quality after Yoga Nidra intervention (t=3.720, p=0.002).

D’ Cunha et al. investigated the effectiveness of Yoga Nidra in reducing stress among women undergoing curative radiotherapy for cervical cancer [8]. This prospective two-arm study included 48 participants who were randomly allocated to an experimental group practising Yoga Nidra or a control group receiving standard care. Stress levels were measured before and after the radiation treatment using a stress scale. The results revealed a significant reduction in stress levels in the Yoga Nidra group compared to the control group (mean scores: 64.42 vs. 79.46; p<0.0001), indicating that Yoga Nidra effectively mitigates stress in cervical cancer patients undergoing radiotherapy.

Nuzhath et al. assessed the combined effects of Yoga Nidra and Pranayama (a yogic breathing technique) on anxiety and depression in women with cervical cancer undergoing standard care [9]. Seventy patients were randomly assigned to an experimental group that received 30-minute sessions of Yoga Nidra and Pranayama twice daily, five days a week for six weeks, or a control group that received only standard care. Anxiety and depression were measured using the Hospital Anxiety and Depression Scale (HADS) at baseline and in the second, fourth, and sixth weeks. The findings revealed a significant reduction in anxiety and depression levels in the experimental group compared with the control group (p ≤ 0.05).

Additionally, a comprehensive review by Musto and Vallerand synthesised findings from 29 studies on Yoga Nidra across various medical conditions, including cancer [2]. This review elucidated the positive effects of reducing stress and improving mood, sleep, and overall well-being, although heterogeneity was observed in study designs and outcomes. While extant studies have demonstrated the efficacy of Yoga Nidra in enhancing sleep quality and mitigating stress and psychological distress among cancer patients, they are not without limitations. Most studies have small sample sizes, short follow-up periods, and inconsistencies in the study design. Furthermore, variations in the Yoga Nidra protocols present challenges in generalizing the results. These limitations underscore the necessity for larger, more rigorous studies with standardized protocols to validate the findings and assess their long-term impacts.

The unique advantages of Yoga Nidra over traditional Yoga

Yoga Nidra, a highly accessible practice requiring minimal physical effort, is particularly well-suited for cancer patients undergoing intensive treatments or those with significant physical constraints. The practice's focus on deep relaxation and inner awareness helps alleviate psychological distress, including anxiety and depression, which are common among cancer patients because of the emotional and physical toll of their illness. By creating a tranquil environment, Yoga Nidra assists in addressing these mental health issues, thereby enhancing psychological resilience. Moreover, this technique is effective in improving sleep quality, which is often compromised by pain, side effects of medication, and psychological stress. Yoga Nidra helps minimize sleep disturbances by promoting relaxation and reducing anxiety, leading to more restorative rest. This comprehensive approach not only addresses the psychological and emotional needs of cancer patients but also supports their overall well-being by combining both psychological and physiological care aspects, thus offering a holistic complementary therapy in cancer management.

Further studies should examine larger and more diverse groups to validate the promising outcomes observed in smaller trials. Furthermore, extended study periods would be beneficial in determining the long-term advantages of Yoga Nidra. More rigorous methodologies are required to standardize the practice for specific cancer patient groups, ensuring uniformity in its application and quantifiable results.

Conclusion

While evidence supporting Yoga Nidra in cancer care remains emergent, preliminary findings indicate its potential as a promising complementary therapy for managing psychological distress and sleep disturbances. Given its non-invasive, cost-effective, and accessible nature, Yoga Nidra may constitute a valuable addition to supportive care protocols for cancer patients. Considering the encouraging evidence for Yoga Nidra as complementary therapy, integrating it into standard oncology care may improve patients' psychological and emotional well-being during treatment. Pilot programs within oncology departments could provide valuable insights into their practical application and benefits, potentially leading to broader adoption across cancer care settings.
